# Association Between ABO Blood Group, COVID-19 Status, and Early Respiratory Outcomes in Acute Pulmonary Embolism: An Interaction Analysis

**DOI:** 10.3390/jcdd13050212

**Published:** 2026-05-14

**Authors:** Abdulkader Jamal Eddin, Stefan-Iulian Stanciugelu, Arnaldo Dario Damian, Diana Nitusca, Oana Elena Tunea, Ioana Monica Mozos

**Affiliations:** 1 Doctoral School, “Victor Babeş” University of Medicine and Pharmacy, 300041 Timişoara, Romania; abdulkader.jamal-eddin@umft.ro; 2 Center for Translational Research and Systems Medicine, “Victor Babeş” University of Medicine and Pharmacy, 300173 Timişoara, Romania; 3 Gastroenterology and Hepatology Clinic, “Pius Brînzeu” County Emergency Clinical Hospital, 300723 Timişoara, Romania; 4 Orthopedics II Research Center, “Pius Brînzeu” County Emergency Clinical Hospital, 300723 Timişoara, Romania; 5 Orthopedics Clinic II, “Pius Brînzeu” County Emergency Clinical Hospital, 300723 Timişoara, Romania; 6 Neurology Clinic II, “Pius Brînzeu” County Emergency Clinical Hospital, 300723 Timişoara, Romania; dariodamian996@gmail.com; 7 Department of Biochemistry and Pharmacology, “Victor Babeş” University of Medicine and Pharmacy, Pta Eftimie Murgu Nr. 2, 300041 Timisoara, Romania; nitusca.diana@umft.ro; 8 Center for Complex Networks Science, “Victor Babeş” University of Medicine and Pharmacy, Pta Eftimie Murgu Nr. 2, 300041 Timisoara, Romania; 9 Advanced Research Center in Cardiovascular Pathology and Hemostaseology, “Victor Babeş” University of Medicine and Pharmacy, 300173 Timişoara, Romania; ancusa.oana@umft.ro; 10 Internal Medicine Clinic, Emergency Municipal Clinical Hospital, 300041 Timişoara, Romania; 11 Department of Functional Sciences-Pathophysiology, “Victor Babeş” University of Medicine and Pharmacy, 300173 Timişoara, Romania

**Keywords:** ABO blood group, pulmonary embolism, COVID-19, venous thromboembolism, endothelial dysfunction, invasive mechanical ventilation, Pulmonary Embolism Severity Index

## Abstract

The influence of COVID-19 infection on the association between ABO blood groups and early outcomes in patients with acute pulmonary embolism (PE) remains uncertain. We conducted a retrospective, single-center cohort study including adult patients admitted with a first episode of acute pulmonary embolism (PE). The interaction between ABO blood group (non-O vs. O) and COVID-19 status was evaluated using multivariable logistic regression models adjusted for PE severity assessed by the Pulmonary Embolism Severity Index (PESI). A total of 211 patients were included, of whom 95 (45.0%) were COVID-19-positive. Among COVID-19-positive patients, non-O blood groups were associated with significantly higher odds of invasive mechanical ventilation (IMV) compared with group O (adjusted odds ratio [aOR] 12.87, 95% CI 4.17–39.75), whereas no association was observed among COVID-19–negative patients (aOR 1.20, 95% CI 0.45–3.23). No interaction was identified for 24 h mortality (*p* = 0.721) or systemic thrombolysis (*p* = 0.306). Higher PESI class was independently associated with an increased risk of adverse outcomes. ABO blood group modified the association between COVID-19 infection and early respiratory outcomes in acute PE. These findings suggest a potential role of ABO-related differences in coagulation and endothelial biology in the clinical expression of COVID-associated PE and should be interpreted as hypothesis-generating.

## 1. Introduction

Pulmonary embolism (PE) is a major manifestation of venous thromboembolism and a significant cause of cardiovascular morbidity and mortality. Although contemporary risk stratification integrates clinical parameters, imaging findings, and biomarkers to identify patients at risk of early deterioration, interindividual variability in clinical severity and early progression suggests that underlying biological determinants may influence disease severity [1,2,3,4,5,6,7,8,9].

ABO blood group is an established genetic risk factor for venous thromboembolism. Individuals with non-O blood groups exhibit higher circulating levels of von Willebrand factor (vWF) and factor VIII due to reduced proteolytic clearance, resulting in a prothrombotic phenotype and a 1.5- to 2-fold increased risk of VTE compared with blood group O. While the association between ABO blood group and VTE incidence is well documented, its impact on the clinical severity and short-term outcomes of acute PE remains incompletely characterized [10,11,12,13,14,15,16].

SARS-CoV-2 infection is associated with endothelial activation, systemic inflammation, and dysregulated coagulation, with marked elevations in vWF and increased thrombotic risk. Given that both non-O blood group status and COVID-19 infection modulate vWF-mediated and endothelial pathways, a biologically plausible interaction could amplify thrombotic severity in patients presenting with acute PE [17,18,19,20,21,22,23].

Despite these mechanistic links, data evaluating potential effect modification between ABO blood group and COVID-19 infection in patients with established acute PE are limited. Whether COVID-19 amplifies the prothrombotic phenotype associated with non-O blood groups, thereby influencing early deterioration, remains unknown.

The present study aimed to evaluate whether COVID-19 status modifies the association between ABO blood group and early in-hospital outcomes among patients admitted with a first episode of acute pulmonary embolism.

## 2. Materials and Methods

### 2.1. Study Design and Setting

**Figure 1:**
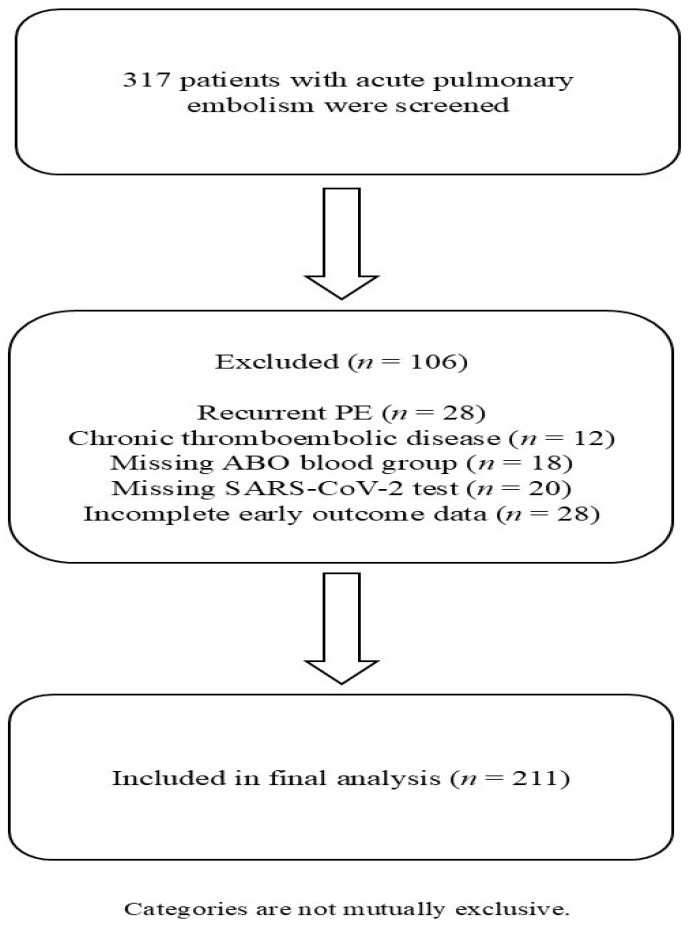
Study flow diagram. Flowchart illustrating patient selection, including reasons for exclusion. Categories are not mutually exclusive.

This retrospective, observational, single-center cohort study included consecutive adult patients meeting predefined inclusion criteria who were admitted with a first episode of acute pulmonary embolism (PE) to the Municipal Hospital of Timișoara, Romania, between 1 April 2020, and 3 June 2023. The study flow diagram is presented in Figure 1.

During the study period, patients hospitalized for acute PE underwent systematic screening for SARS-CoV-2 infection at admission according to institutional protocols. Based on the admission screening result, patients were classified as COVID-19-positive or COVID-19-negative. The study was approved by the Ethics Committee of the Victor Babeș University of Medicine and Pharmacy Timișoara (Approval No. 96/04.10.2021, revised 26 October 2025), and was conducted in accordance with the Declaration of Helsinki. Owing to the retrospective nature of the study and the use of anonymized data, the requirement for informed consent was waived.

The objective was to assess whether ABO blood group modifies the association between COVID-19 status and early in-hospital outcomes in acute pulmonary embolism.

Inclusion Criteria: Patients were eligible if they met all of the following criteria: age ≥ 18 years; acute PE confirmed by computed tomography pulmonary angiography (CTPA); clinical presentation compatible with acute PE; first documented episode of PE in the patient’s medical history, verified using national and hospital databases; availability of documented SARS-CoV-2 testing at admission using RT-PCR.

Exclusion Criteria: Patients were excluded if they had a history of recurrent PE; chronic thromboembolic disease, including previously diagnosed chronic thromboembolic pulmonary hypertension (CTEPH); absence of documented ABO blood group in hospital medical records; absence of documented SARS-CoV-2 test result at admission; or incomplete documentation of early in-hospital outcomes.

After applying inclusion and exclusion criteria, 211 patients were included in the final analysis, while 106 were excluded.

### 2.2. Outcome Definitions

ABO blood group was coded a priori as non-O (A/B/AB) versus O. Early in-hospital outcomes were defined as events occurring within the first 24 h after admission. The focus on 24 h outcomes was chosen to capture early clinical deterioration and acute respiratory failure occurring shortly after hospital admission. The primary endpoint was 24 h in-hospital mortality, defined as death from any cause occurring within 24 h of hospital admission for acute pulmonary embolism. Secondary endpoints included requirement for systemic thrombolysis within 24 h of admission, defined as administration of intravenous fibrinolytic therapy for acute PE, and requirement for invasive mechanical ventilation (IMV) within 24 h of admission, defined as endotracheal intubation with mechanical ventilatory support. All outcomes were ascertained from medical records and hospital documentation.

### 2.3. Data Collection and Variables

Data were extracted from electronic and paper medical records, hospital discharge summaries, and the institutional laboratory database. The ABO blood group was obtained from the hospital laboratory information system. Data extraction was independently performed by two investigators, with discrepancies resolved by consensus. No imputation of missing data was performed.

Acute PE was confirmed by contrast-enhanced multidetector CTPA performed using standardized pulmonary embolism acquisition protocols on Siemens multidetector CT systems (Somatom Definition Edge and Somatom go platforms; Siemens Healthineers, Erlangen, Germany). Image interpretation and thrombus localization were performed by board-certified radiologists as part of routine clinical practice.

Collected variables included demographic characteristics (age and sex) and comorbidities documented prior to admission, including chronic venous insufficiency, chronic obstructive pulmonary disease, pulmonary hypertension, cancer, heart failure, hypertension, atrial fibrillation, prior myocardial infarction, prior stroke, hematologic diseases, diabetes mellitus, and obesity. Hematologic diseases were defined as previously diagnosed disorders of the hematopoietic system, including chronic anemia, myeloproliferative or myelodysplastic syndromes, leukemia, lymphoma, inherited or acquired coagulopathies, and other chronic hematologic conditions documented in the medical record. Obesity was defined as a documented diagnosis or body mass index ≥ 30 kg/m^2^.

Collected clinical variables included mortality, Pulmonary Embolism Severity Index (PESI) score at admission, requirement for systemic thrombolysis, and requirement for invasive mechanical ventilation. COVID-19 status was defined by RT-PCR testing performed at admission. PESI was extracted from admission documentation and analyzed descriptively as a continuous variable and according to established risk classes (I–V). Admission to the intensive care unit (ICU) was not used as an outcome because it may reflect institutional policies and resource availability rather than disease severity. Patients were followed throughout hospitalization until discharge, transfer, or death, although the present analysis focused on outcomes occurring within the first 24 h after admission.

Laboratory analyses were performed in the hospital’s central laboratory using standardized automated platforms, including hematology analyzers (Sysmex XN-1000, Sysmex Corporation, Kobe, Japan; Nihon Kohden MEK-9100, Nihon Kohden Corporation, Tokyo, Japan), coagulation analyzers (Sysmex CS-2500, Sysmex Corporation, Kobe, Japan), and biochemistry analyzers (Cobas 6000 c501, Roche Diagnostics, Mannheim, Germany; Dimension EXL 200, Siemens Healthineers, Erlangen, Germany). All assays were conducted according to the manufacturers’ instructions and internal quality control procedures in place during the study period. SARS-CoV-2 infection was determined by RT-PCR testing of nasopharyngeal swab samples performed at admission.

### 2.4. Statistical Analysis

Continuous variables were assessed for distribution and are presented as mean ± standard deviation (SD) for approximately normally distributed variables or median with interquartile range (IQR) otherwise. Categorical variables are presented as absolute counts and percentages. Between group comparisons were performed using the independent samples *t*-test or Mann–Whitney U test for continuous variables, as appropriate, and the χ^2^ test or Fisher’s exact test for categorical variables depending on expected cell counts. Analyses were conducted using complete case data; no imputation of missing values was performed. All tests were two-sided, with statistical significance defined as *p* < 0.05 for the primary endpoint. Statistical analyses were performed using Python 3.14.0 with the matplotlib and statsmodels libraries.

ABO blood group was analyzed primarily as O versus non-O (A, B, AB). The interaction between ABO blood group and COVID-19 status was evaluated using multivariable logistic regression models including the main effects of COVID-19 status and ABO group and a multiplicative interaction term (COVID-19 × non-O blood group). Multivariable logistic regression models included COVID-19 status, ABO blood group (non-O vs. O), and their interaction term, and were adjusted for pulmonary embolism severity using the Pulmonary Embolism Severity Index (PESI) class. Statistical interaction was assessed using the Wald test for the interaction term (reported as the “*p* for interaction”).

Separate models were constructed for each clinical outcome. Multivariable models were adjusted for pulmonary embolism severity using the Pulmonary Embolism Severity Index (PESI) class, modeled as an ordinal variable. Additional adjustment for individual comorbidities was not performed because several of these variables are components of the PESI score and their simultaneous inclusion could introduce collinearity. Covariates were selected a priori based on clinical relevance. Pulmonary embolism severity was accounted for using the Pulmonary Embolism Severity Index (PESI), a validated composite score. Univariate screening was not used for variable selection in order to avoid data-driven bias, overfitting, and model instability, particularly in the context of interaction analyses.

Firth penalized logistic regression was used for the primary endpoint and for thrombolysis models when separation was observed. Standard logistic regression was used for invasive mechanical ventilation models. Results are reported as adjusted odds ratios (aORs) with 95% confidence intervals (CIs). For interpretability, stratum specific estimates for the association between non-O blood group and each endpoint were derived within COVID-negative and COVID-positive strata from the fitted interaction models. Adjusted predicted probabilities were additionally computed for the four exposure groups (O/COVID−, non-O/COVID−, O/COVID+, non-O/COVID+). Sensitivity analyses evaluated ABO as a four-level categorical variable (O, A, B, AB) using O as the reference category.

To assess the robustness of regression coefficient estimates and the potential impact of model instability, bootstrap resampling with 1000 iterations was performed as a supplementary sensitivity analysis. In each iteration, the full dataset was resampled with replacement at the patient level while preserving the original sample size, and the regression model was refitted. Bootstrap mean coefficient estimates and bootstrap-derived 95% confidence intervals were obtained from the empirical distribution of the resampled coefficients. The original model coefficients were compared with the bootstrap mean estimates to assess potential bias related to model instability.

## 3. Results

A total of 317 patients with acute pulmonary embolism were screened during the study period. After applying inclusion and exclusion criteria, 211 patients were included in the final analysis and 106 were excluded. Among included patients, 95 (45.0%) were COVID-19-positive and 116 (55.0%) were COVID-19-negative. The distribution of ABO blood groups in the study cohort was as follows: O (*n* = 54, 25.6%), A (*n* = 88, 41.7%), B (*n* = 41, 19.4%), and AB (*n* = 28, 13.3%). Baseline characteristics according to COVID-19 status are presented in Table 1.

Early in-hospital outcomes according to COVID-19 status are presented in Table 2. Patients with COVID-19 had significantly higher rates of 24 h mortality and higher requirement for invasive mechanical ventilation compared with COVID-19-negative patients. The use of systemic thrombolysis within the first 24 h was also more frequent among COVID-19-positive patients.

Crude event rates according to combined ABO blood group and COVID-19 exposure groups are presented in Table 3. The highest rates of invasive mechanical ventilation were observed among patients with non-O blood groups and COVID-19 infection.

Multivariable logistic regression models including an interaction term between ABO blood group (non-O vs O) and COVID-19 infection, adjusted for PESI class, are presented in Table 4. A significant interaction between ABO blood group and COVID-19 infection was observed for invasive mechanical ventilation (*p* for interaction = 0.002). Among COVID-19-positive patients, non-O blood groups were associated with substantially higher odds of invasive mechanical ventilation compared with group O (aOR 12.87, 95% CI 4.17–39.75), whereas no association was observed among COVID-19-negative patients (aOR 1.20, 95% CI 0.45–3.23). No statistically significant interaction between ABO blood group and COVID-19 infection was observed for 24 h mortality (*p* for interaction = 0.721) or systemic thrombolysis (*p* for interaction = 0.306). In all models, higher PESI class was independently associated with increased odds of adverse outcomes.

**Figure 2:**
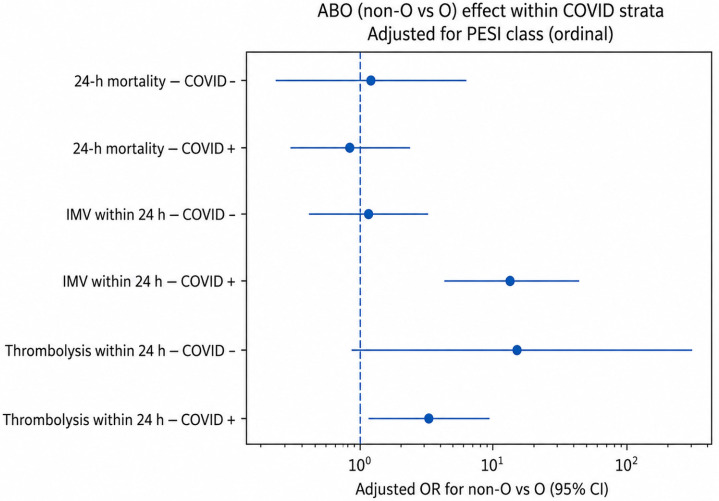
Adjusted association of non-O versus O blood group with early in-hospital outcomes within COVID-negative and COVID-positive strata. Estimates are adjusted for PESI class (ordinal I–V) and derived from models including an ABO × COVID interaction term. Points represent adjusted odds ratios and horizontal lines represent 95% confidence intervals.

Figure 2 illustrates the adjusted association between non-O blood group and early outcomes within COVID-negative and COVID-positive strata.

Sensitivity analyses modeling ABO as a four-level categorical variable (O, A, B, AB) yielded similar results and did not materially change the interaction estimates.

As a supplementary sensitivity analysis using discharge-based in-hospital mortality, death occurred in 12/116 (10.3%) COVID-negative patients and 39/95 (41.1%) COVID-positive patients (*p* < 0.001). In multivariable Firth logistic regression models adjusted for PESI class, no significant interaction between COVID-19 status and non-O blood group was observed for in-hospital mortality (interaction aOR~0.68, 95% CI crossing unity; *p* for interaction non-significant).

To assess coefficient stability, bootstrap resampling with 1000 iterations was performed for the regression models. For the invasive mechanical ventilation model, the bootstrap mean estimate for the COVID × non-O interaction term was similar to the original coefficient (original beta 2.371; bootstrap mean beta 2.450), and the bootstrap-derived 95% confidence interval remained above the null value (0.879 to 3.970), supporting the robustness of this interaction despite limited precision. For mortality, bootstrap analyses confirmed broad uncertainty around the interaction estimate.

## 4. Discussion

In this retrospective cohort study including 211 patients hospitalized with acute pulmonary embolism, we evaluated whether ABO blood group modifies the association between COVID-19 infection and early in-hospital outcomes. The relatively high proportion of COVID-19-positive patients and the elevated rates of early mortality and invasive mechanical ventilation observed in this cohort likely reflect the inclusion of a high-risk hospitalized population during the COVID-19 pandemic. These rates exceed those reported in large registries such as the RIETE registry, where short-term mortality is substantially lower. Similarly, rates of invasive mechanical ventilation in contemporary pulmonary embolism cohorts are considerably lower than those observed in the present study. This discrepancy reflects the specific clinical context of the present study and limits the generalizability of the findings to broader, non-pandemic PE populations. A significant interaction between ABO blood group and COVID-19 infection was observed for invasive mechanical ventilation. Among COVID-19-positive patients, non-O blood groups were associated with substantially higher odds of invasive mechanical ventilation compared with group O, whereas no association was observed among COVID-19-negative patients. No interaction was identified for 24 h mortality or systemic thrombolysis despite the established prothrombotic effects of both non-O blood groups and SARS-CoV-2 infection [17,18,19,20,21,22,23]. These findings suggest that ABO-related thrombotic mechanisms may preferentially contribute to pulmonary microvascular dysfunction and respiratory deterioration rather than directly influencing early mortality. Early endpoints may be particularly relevant in the context of COVID-19-associated pulmonary vascular dysfunction, where rapid clinical deterioration can occur within the first hours of hospitalization. In all models, higher PESI class was independently associated with an increased risk of adverse outcomes.

Because 24 h mortality is an unconventional endpoint in acute pulmonary embolism studies, we additionally evaluated in-hospital mortality based on discharge data. This supplementary analysis did not identify a significant interaction between ABO blood group and COVID-19 status for mortality, consistent with the primary findings. Bootstrap resampling further demonstrated that the interaction observed for invasive mechanical ventilation remained directionally robust, although the wide confidence intervals reflect limited precision.

The hypothesis of effect modification is biologically plausible. Non-O blood groups are associated with higher circulating levels of von Willebrand factor (vWF) and factor VIII, partly due to reduced proteolytic clearance of vWF in individuals lacking the O antigen. Elevated vWF levels promote platelet adhesion, thrombin generation, and endothelial activation, mechanisms that increase the risk of venous thromboembolism. Consistent with these mechanisms, non-O blood groups have repeatedly been associated with a higher incidence of deep vein thrombosis and pulmonary embolism [10,11,12,13,14,15,16].

SARS-CoV-2 infection induces a distinct prothrombotic state characterized by endothelial activation, increased release of von Willebrand factor, platelet activation, and dysregulated coagulation. Severe COVID-19 has been associated with markedly elevated vWF levels, markers of endothelial injury, and pulmonary microvascular thrombosis. Because both non-O blood group status and SARS-CoV-2 infection influence vWF-mediated pathways and endothelial dysfunction, a synergistic interaction may amplify thrombotic and microvascular injury in patients presenting with acute pulmonary embolism [17,18,19,20,21,22,23,24,25].

These mechanisms may contribute to pulmonary vascular obstruction beyond the embolic clot burden detected on imaging and may partly explain the higher risk of respiratory deterioration observed in COVID-19-positive patients.

To our knowledge, few studies have specifically evaluated the interaction between ABO blood group and COVID-19 infection in determining the clinical severity of acute pulmonary embolism. The absence of a significant interaction for mortality may reflect the multifactorial determinants of early mortality in acute pulmonary embolism, which are largely captured by established severity indices such as PESI.

Our study has several methodological strengths. First, it included a consecutive cohort of patients with objectively confirmed acute pulmonary embolism, reducing selection bias and improving cohort comparability. COVID-19 status was determined systematically at admission using RT-PCR testing, minimizing the risk of exposure misclassification. Early outcomes were predefined within a 24 h window, which limited heterogeneity related to differences in subsequent in-hospital management. Multivariable models were prespecified and adjusted for clinically relevant covariates without automated variable selection, improving transparency and reproducibility. In addition, the use of Firth penalized logistic regression for mortality and thrombolysis addressed sparse event data and quasi-separation, thereby improving the stability of the estimated effects. Importantly, the study design directly evaluated effect modification through interaction modeling rather than relying solely on subgroup analyses.

This study also has several limitations that should be acknowledged. First, it was a single-center retrospective study, which may limit generalizability and leaves the possibility of residual confounding despite multivariable adjustment. Second, the sample size and event distribution limited statistical power for detecting interaction effects, particularly for early mortality. Interaction analyses typically require larger sample sizes than main effect analyses, and the present study may therefore be underpowered to detect more modest effect modification. The magnitude of the observed association between non-O blood groups and invasive mechanical ventilation should also be interpreted cautiously given the relatively small subgroup sizes and wide confidence intervals. In addition, changes in SARS-CoV-2 variants and evolving clinical management strategies during the study period may have influenced outcomes. These findings should therefore be interpreted cautiously, as the precision of the estimates is limited and model stability cannot be fully ensured. Vaccination status, inflammatory biomarkers, and longitudinal laboratory parameters were not available, precluding a more detailed mechanistic exploration of endothelial activation and coagulation pathways. Importantly, objective measures of COVID-19 severity were not consistently available, and therefore residual confounding related to the severity of SARS-CoV-2 infection cannot be excluded and may have influenced early respiratory outcomes. ABO blood group was analyzed primarily as a binary exposure in the main models, which may obscure subtle subtype-specific effects. Finally, outcomes were restricted to the first 24 h of hospitalization and therefore do not capture later clinical deterioration. The use of 24 h outcomes may limit comparability with major clinical trials that report longer-term endpoints such as 7-day or 30-day mortality, and therefore these findings should be interpreted as reflecting very early clinical deterioration rather than overall prognosis. Therefore, the present findings should be considered hypothesis-generating and require confirmation in larger, prospective studies.

## 5. Clinical Implications

The findings of this study suggest that ABO blood group, particularly non-O status, may contribute to early respiratory deterioration in patients with acute pulmonary embolism in the context of COVID-19. Although these results should be interpreted cautiously, they raise the possibility that ABO blood group could serve as an additional biological marker in early risk stratification. The identification of patients at higher risk of requiring invasive mechanical ventilation may support closer monitoring and timely escalation of care. Further studies are needed to validate these findings and to determine their potential role in clinical decision-making.

## 6. Conclusions

In this cohort of patients hospitalized with acute pulmonary embolism, ABO blood group modified the association between COVID-19 infection and early respiratory outcomes. Non-O blood groups were associated with substantially higher odds of invasive mechanical ventilation among COVID-19-positive patients, whereas no such association was observed among COVID-19-negative patients. No interaction between ABO blood group and COVID-19 infection was detected for early mortality or systemic thrombolysis. These findings suggest that ABO-related differences in coagulation and endothelial biology may contribute to pulmonary vascular dysfunction and influence the clinical expression of COVID-associated pulmonary embolism. Further studies are warranted to confirm these observations and to clarify their potential clinical implications.

## Figures and Tables

**Table 1:** Baseline characteristics of acute PE patients, stratified by COVID status.

Variable	COVID-Negative (*n* = 116)	COVID-Positive (*n* = 95)
Age, years	69 [60–79]	72 [63.5–80.5]
Male sex	60 (51.7%)	60 (63.2%)
PESI class		
I	21 (18.1%)	9 (9.5%)
II	21 (18.1%)	7 (7.4%)
III	32 (27.6%)	14 (14.7%)
IV	22 (19.0%)	14 (14.7%)
V	20 (17.2%)	51 (53.7%)
ABO blood group		
O	26 (22.4%)	28 (29.5%)
A	49 (42.2%)	39 (41.1%)
B	27 (23.3%)	14 (14.7%)
AB	14 (12.1%)	14 (14.7%)
Comorbidities		
Cancer	25 (21.6%)	28 (29.5%)
Chronic lung disease	20 (17.2%)	19 (20.0%)
Heart failure	72 (62.1%)	71 (74.7%)
Chronic venous insufficiency	31 (26.7%)	11 (11.6%)
Pulmonary hypertension	58 (50.0%)	48 (50.5%)
Hypertension	75 (64.7%)	70 (73.7%)
Atrial fibrillation	17 (14.7%)	22 (23.2%)
Prior myocardial infarction	9 (7.8%)	11 (11.6%)
Prior stroke	22 (19.0%)	17 (17.9%)
Hematologic disease	32 (27.6%)	30 (31.6%)
Diabetes mellitus	18 (15.5%)	13 (13.7%)
Obesity	46 (39.7%)	24 (25.3%)
Chronic kidney disease	36 (31.0%)	25 (26.3%)

For PESI class and ABO, the *p* value refers to the overall comparisons across categories.

**Table 2:** Early in-hospital outcomes of acute PE patients according to COVID-19 status.

Outcome	COVID-Negative (*n* = 116)	COVID-Positive (*n* = 95)	*p* Value
24 h mortality	12 (10.3%)	32 (33.7%)	0.002
Invasive mechanical ventilation	46 (39.7%)	66 (69.5%)	<0.001
Systemic thrombolysis	26 (22.4%)	34 (35.8%)	0.031

Values are presented as *n* (%). *p* values were calculated using the χ^2^ test or Fisher’s exact test.

**Table 3:** Early in-hospital outcomes according to combined ABO blood group and COVID-19 exposure.

Outcome	O/COVID− (*n* = 26)	Non-O/COVID− (*n* = 90)	O/COVID+ (*n* = 28)	Non-O/COVID+ (*n* = 67)
24 h mortality	2 (7.7%)	10 (11.1%)	12 (42.9%)	26 (38.8%)
Invasive mechanical ventilation	8 (30.8%)	33 (36.7%)	12 (42.9%)	59 (88.1%)
Systemic thrombolysis	0 (0.0%)	20 (22.2%)	7 (25.0%)	33 (49.3%)

**Table 4:** Interaction between ABO blood group and COVID-19 for early outcomes (adjusted for PESI class).

Outcome	Model	Interaction aOR (COVID × Non-O)	*p* for Interaction	Non-O vs O aOR in COVID−	Non-O vs O aOR in COVID+	PESI Class aOR per Class
24 h mortality	Firth logistic	0.71 (0.11–4.54)	0.721	1.18 (0.25–5.62)	0.84 (0.31–2.26)	2.47 (1.69–3.61)
IMV within 24 h	Logistic	10.71 (2.38–48.09)	0.002	1.20 (0.45–3.23)	12.87 (4.17–39.75)	1.69 (1.31–2.18)
Thrombolysis within 24 h	Firth logistic	0.2 (0.01–4.38)	0.306	15.84 (0.85–294.64)	3.15 (1.14–8.71)	1.79 (1.35–2.38)

Models include COVID-19 status, ABO blood group (non-O vs O), the interaction term (COVID-19 × non-O), and pulmonary embolism severity assessed by PESI class modeled as an ordinal variable. Interaction *p* values were obtained from the Wald test for the multiplicative interaction term. Firth penalized logistic regression was used for outcomes with sparse events.

## Data Availability

The data supporting the findings of this study are available from the corresponding author upon reasonable request, in accordance with institutional and ethical regulations.

## Abbreviations

The following abbreviations are used in this manuscript:

ABO	ABO blood group system
aOR	Adjusted odds ratio
CI	Confidence interval
COVID-19	Coronavirus disease 2019
CTEPH	Chronic thromboembolic pulmonary hypertension
CTPA	Computed tomography pulmonary angiography
ICU	Intensive care unit
IMV	Invasive mechanical ventilation
PE	Pulmonary embolism
PESI	Pulmonary Embolism Severity Index
RT-PCR	Reverse transcription polymerase chain reaction
SARS-CoV-2	Severe acute respiratory syndrome coronavirus 2
VTE	Venous thromboembolism
vWF	Von Willebrand factor
